# Safety of antidepressants commonly used in 6–17-year-old children and adolescents: A disproportionality analysis from 2014–2023 on the basis of the FAERS database

**DOI:** 10.1371/journal.pone.0330025

**Published:** 2025-08-13

**Authors:** Yan Zhou, Yun Lu, Yu Wu, Wenqiang Kong, Lu Zhou, Xirui Guo, Hua Wei, Hongju Wang, Fangqing Xie, Shihao Yan, Fang Yang, Chun Liu, Qinchuan Li, Na Li, Ya Zou, Jia Chen

**Affiliations:** 1 Department of Pharmacy, Chengdu Second People’ s Hospital, Chengdu, Sichuan, China; 2 Department of Pharmacy, Zigong First People’s Hospital, Zigong, Sichuan, China; 3 Department of Pharmacy, Central People’ s Hospital of Zhanjiang, Zhanjiang, Guangdong, China; 4 Department of Pharmacy, Chengdu Jinniu District People’s Hospital, Chengdu, Sichuan, China; Northwestern University Feinberg School of Medicine, UNITED STATES OF AMERICA

## Abstract

**Background:**

Depression is a common mental disorder in children and adolescents, and antidepressants are widely used for treatment. This study aimed to explore and analyze adverse events (AEs) associated with antidepressant use in this population, providing insights for safety assessment.

**Methods:**

This study extracted AE reports from the US FDA Adverse Event Reporting System (FAERS) for children and adolescents aged 6–17 years from 2014–2023, with fluoxetine, escitalopram, and sertraline as the primary suspected drugs. The study used the International Dictionary of Medical Terminology (MedDRA) to encode and classify AEs, and employed the reporting odds ratio (ROR) and proportional reporting ratio (PRR) methods for data mining.

**Results:**

The FAERS database included 9,845 AE reports involving fluoxetine, escitalopram, and sertraline among children and adolescents aged 6–17 years. After removing duplicates, the analysis yielded 1,604, 352, and 571 AEs for fluoxetine, escitalopram, and sertraline, respectively. The study population demonstrated a sex distribution with approximately twice as many female patients as male patients, with most patients aged 12–17 years. At the system organ class (SOC) level, the AEs predominantly involved psychiatric disorders and nervous system disorders. Within the psychiatric disorders category at the high level group term (HLGT) level, the most frequently reported AE signals across all three medications were suicidal and self-injurious behaviors (not elsewhere classified, hereinafter referred to as NEC) and anxiety disorders and symptoms; For nervous system disorders, the predominant AE signals were neurological disorders (NEC) and movement disorders (including parkinsonism). At the Preferred Term (PT) level, the three antidepressants demonstrated similar AEs, including various suicidal and self-injurious behaviors, intentional overdose, neurological disorders (including serotonin syndrome, extravertebral reactions, etc) and prolonged QT. The typical AEs of fluoxetine included decreased appetite, insomnia, and urinary retention. Escitalopram was associated with additional AEs of sexual dysfunction and toxic epidermal necrolysis, whereas sertraline demonstrated unique associations with headache and rhabdomyolysis.

**Conclusion:**

This study may offer further evidence regarding AEs associated with commonly prescribed antidepressants in children and adolescents. Overall, the findings are mostly consistent with the AEs recorded in the packaging instructions and previous reports. However, the study also identified previously unreported AEs and highlighted variations in the AE profiles across different demographic groups. As the causal relationships between these medications and the observed AEs remain to be fully elucidated, additional research is warranted to confirm these findings.

## Introduction

Depression is a common mental disorder characterized by persistent sadness and loss of interest, and is one of the most prevalent, disabling, and costly diseases worldwide [1]. As of 2013, major depression was the second leading cause of disability worldwide [2]. It affects approximately 3% of prepubertal children and 6% of postpubertal adolescents [3,4], making its treatment in this population a critical public health issue.

Treatment strategies include psychotherapy, such as cognitive‒behavioral therapy and interpersonal therapy, which are traditionally dominant for children and adolescents [1,5]. However, for moderate to severe cases or those with comorbidities, evidence-based drug therapy remains an essential option. Notably, among antidepressants, only fluoxetine and escitalopram have received FDA approval for children and adolescents. Fluoxetine is indicated for patients aged ≥8 years, whereas escitalopram is approved for those ≥12 years. Current evidence suggested that these medications, particularly fluoxetine, remain the preferred therapeutic options for this population [6,7]. When fluoxetine and escitalopram prove ineffective or intolerable, alternative antidepressants such as sertraline may be considered. However, the use of other agents, including citalopram, fluvoxamine, and paroxetine in pediatric depression treatment is limited [8,9], resulting in significantly lower clinical utilization than the three primary options.

Selective serotonin reuptake inhibitors (SSRIs) represent the primary class of antidepressants for pediatric patients, and serve first-line treatment due to their efficacy and tolerability. In addition to depression, SSRIs demonstrate therapeutic efficacy across multiple psychiatric conditions, including anxiety disorders, obsessive‒compulsive spectrum disorders, trauma-related disorders, and somatic symptom disorders. Currently, antidepressant prescriptions for children and adolescents with depression have shown a marked upward trend [5,10], with standard treatment protocols typically extending 6–12 months beyond symptom resolution [11,12].

Moreover, the widespread utilization and extended treatment duration of antidepressant treatment in children and adolescents have increased concerns regarding drug-related adverse events (AEs), particularly the association between the use of SSRIs and the increased risk of suicide [13]. In May 2007, the FDA mandated a black box warning for antidepressants, highlighting the increased risk of suicide in young adults (18–24 years) during initial treatment [14,15]. Extensive research has identified multiple antidepressant-associated AEs, including cardiovascular toxicity [16,17], serotonin syndrome [18], sexual dysfunction, abnormal bleeding [19–21], hyponatremia [22,23], visual disturbances [24], and rhabdomyolysis [25]. However, current evidence is predominantly derived from adult populations, with limited pediatric clinical trial data available. The potential for SSRIs to induce rare or severe AEs in pediatric patients remains uncertain. Developmental factors significantly influence the drug response, as children and adolescents possess immature immune regulation and distinct anatomical/physiological characteristics compared with adults [26]. These age-specific pharmacological and pharmacokinetic variations predispose pediatric populations to increased drug toxicity risks.

A recent network meta-analysis extensively evaluated antidepressant efficacy and tolerability in children and adolescents, but provided limited analysis of specific adverse reactions [10]. A prior investigation assessed the safety of fluoxetine in children and adolescents via FDA Adverse Event Reporting System (FAERS) data (2015–2019), but excluded other antidepressants from its analysis [27]. Another FAERS-based study examined SSRIs safety characteristics and potential AEs, although it did not specifically focus on pediatric populations [28]. Notably, there remains a critical lack of systematic analyses utilizing global big data to investigate AE signals in the use of pediatric antidepressant. To address these limitations, the current study employed the FAERS database to conduct a comprehensive evaluation of AEs associated with commonly prescribed antidepressants (fluoxetine, escitalopram, and sertraline) in children and adolescents. Through detailed comparative analysis of AEs, real-world safety profiles, informing clinical practice and enhancing medication safety can be identified.

## Materials and methods

### Research subjects

In our study, we focused on fluoxetine, escitalopram, and sertraline as our primary research subjects. While our initial research scope included all SSRIs [29], preliminary data analysis indicated insufficient AE reports for citalopram, paroxetine, fluvoxamine, and vilazodone to support a comprehensive safety evaluation. Therefore, these four medications were excluded from the final analysis.

### Data source and preprocessing

The FAERS database, a publicly accessible pharmacovigilance database updated quarterly, served as the data source for this real-world study [30]. We conducted a retrospective disproportionality analysis using FAERS data from Q1 2014 to Q4 2023, focusing on antidepressant-related AEs in patients aged 6–17 years. The analysis included only cases where the target antidepressants (fluoxetine, escitalopram, and sertraline) were listed as primary suspects, while “secondary suspicion”, “concurrent medication”, and “interaction” were excluded. AEs were classified via International Dictionary of Medical Terminology (MedDRA,version 27.0), with events coded at the Preferred Term (PT) level and mapped to the system organ class (SOC) and high level group term (HLGT) categories. Following FDA guidelines, we processed the dataset by removing duplicates and standardizing entries. Less frequently reported antidepressants identified during preliminary screening were excluded from the analysis.

### Data analysis

We identified potential AE signals via disproportionality analysis (Table 1), employing both the reporting odds ratio (ROR) and the proportional reporting ratio (PRR) for joint testing (Table 2). This approach evaluates signal characteristics through two distinct dimensions: statistical association strength (signal strength/intensity) and reporting frequency (signal frequency). Signal strength/intensity was quantified through ROR values (95% CI lower limit >1) and PRR values (≥2 with χ^2^ ≥ 4), reflecting the degree of association between the drug and AEs. Signal frequency was quantified by case report counts (≥3 cases), indicating the number of reports in the database where a specific drug is associated with a particular AE. From a clinical perspective, high-intensity signals (e.g., significantly elevated ROR values) may suggest drug-specific safety risks, while high-frequency signals reflect the level of clinical concern for particular adverse events. We ultimately classified signals meeting both algorithmic thresholds as antidepressant-associated AEs. Significant PT-level signals were selected for further analysis.

**Table 1:** Fourfold table for disproportionality analyses.

Event groups	Drug used	Other drugs	Sums
Event	a	c	a + c
Other events	b	d	b + d
Sums	a + b	c + d	a + b + c + d

**Table 2:** Computational formula and threshold of signal detection.

Algorithms	Equation	Criteria
ROR	ROR =ad/bc\)	95%CI > 1, N ≥ 3\)
	$95\%CI=e^{ln(ROR)\pm 1.96(1/a+1/b+1/c+1/dhat0.5}\backslash )$	
PRR	PRR =[a(c + d)]/[c(a + b)]\)	PRR≥2, χ2 ≥ 4, N ≥ 3\)
	χ2=[(ad−bchat2](a + b + c + d)/[(a + b)(c +d)(a + c)(b + d)]\)	

The clinical characteristics of the patients, including sex, age, reporting country, and reporter, were analyzed descriptively. Dichotomous variables are expressed as frequencies and percentages, whereas continuous variables are presented as medians and interquartile ranges. Data analysis was performed via Microsoft Excel 2016 (Microsoft, Redmond, WA, USA) and GraphPad Prism 10 (GraphPad Software, CA, USA).

## Results

### Flowchart of reporting

**Fig 1:**
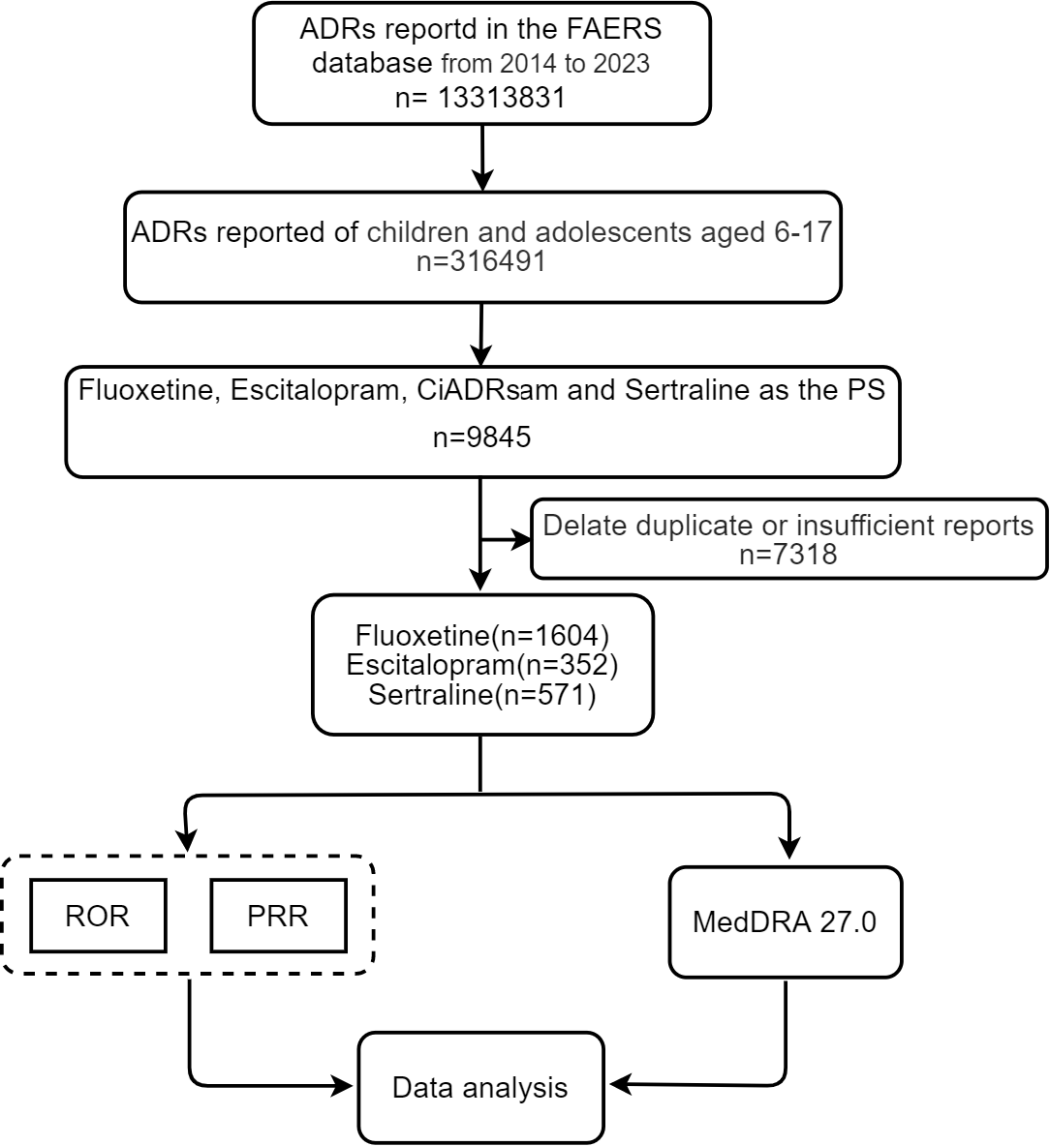
Study flowchart of FAERS reports.

This study analyzed 13,313,831 cases from the FAERS database (2014–2023), including 316,491 reports involving children and adolescents aged 6**–**17 years. Among these, these were 9,845 cases in which fluoxetine, escitalopram, and sertraline were the primary drug. After excluding duplicates and insufficient reports, we identified 1,604 fluoxetine-related, 352 escitalopram-related, and 571 sertraline-related AEs for further analysis. Using the predefined threshold criteria from the aforementioned detection methods, we identified significant AE signals for further analysis. The study design is illustrated in Fig 1.

### Basic characteristics of reported AEs

We analyzed the demographic characteristics, reporters, and geographic distributions of the three drugs. Among all the patients, 61.91% (fluoxetine), 73.23% (escitalopram), and 58.67% (sertraline) were female, whereas 32.54%, 29.54%, and 33.45% were male, respectively. The majority of cases occurred in adolescents aged 12–17 years, followed by those aged 9–11 years, with the lowest proportion in those aged 6–8 years. Most reports originated from healthcare professionals, with the United States reporting the highest number of AEs. The detailed demographic characteristics are presented in Table 3.

**Table 3:** Basic information of AE reports.

Characteristics	Fluoxetine(N = 1604)	Escitalopram(N = 352)	Sertraline(N = 571)
Age (years)			
Mean(SD)	14.47(2.38)	14.78(2.37)	14.01(2.65)
Median(Q1,Q3)	15.00(14-16)	15.00(14-17)	15.00(13-16)
6-8	54(3.37)	13(3.69)	28(4.90)
9-11	127(7.92)	19(5.40)	64(11.21)
12-15	796(49.63)	154(43.75)	284(49.74)
16-17	627(39.09)	166(47.16)	195(34.15)
Gender (n,%)			
Male	522(32.54)	96(29.54)	191(33.45)
Female	993(61.91)	238(73.23)	335(58.67)
Unknown	89(5.55)	18(5.54)	45(7.88)
Type of reporter			
Health professional	1089(67.89)	246(69.89)	318(55.69)
Non health professional	475(29.61)	86(24.43)	237(41.51)
Unknown	40(2.49)	20(5.68)	16(2.80)
Reporting countries (Top five)			
	US 553(34.48)	US 192(54.55)	US 255(44.66)
	GB 276(17.21)	FR 28(7.95)	CN 55(9.63)
	FR 139(8.67)	DE 22(6.25)	FR 36(6.30)
	SE 97(6.05)	DK 19(5.40)	HR 36(6.30)
	DE 94(5.86)	CA 15(4.26)	IT 32(5.60)

N: the number of AEs reports. SD: Standard Deviation; Q1: Lower Quartile; Q3: Upper Quartile; US: United States of America; GB: United Kingdom; FR: France; SE: Swede; DE: Deutschland; DK: Denmark; CA: Canada; CN:China; HR: Croatia; IT: Italy.

### Signal distribution and detection at the SOC and HLGT levels

AEs associated with fluoxetine, escitalopram, and sertraline were categorized according to the SOC of MedDRA 27.0, covering 26 SOC categories from the FAERS database, excluding benign, malignant, and unspecified neoplasms. Three SOC classifications—social circumstances, product issues, and surgical and medical procedures—were omitted. Additionally, AEs related to psychiatric disorders and nervous system disorders were further classified via the HLGT.

**Fig 2:**
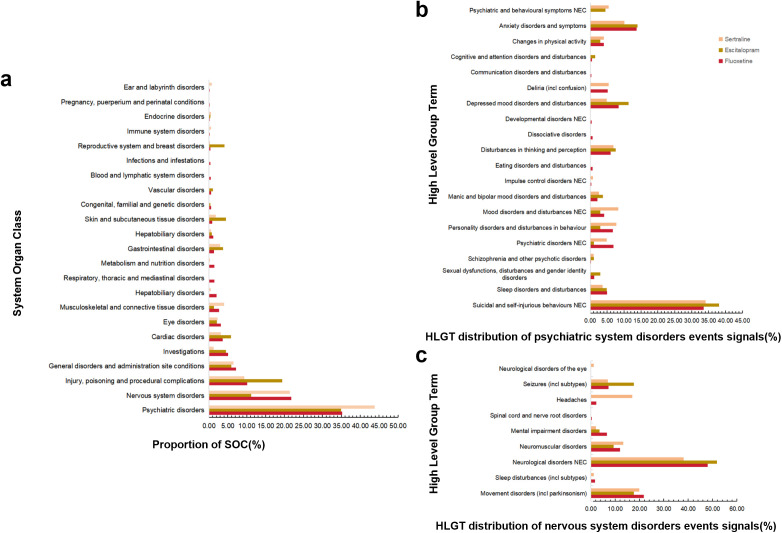
(a) AEs distribution for fluoxetine, escitalopram, and sertraline at the SOC level. (b) HLGT distribution of psychiatric disorders event signals. (c) HLGT distribution of nervous system disorders event signals.

As shown in Fig 2a, among the remaining 23 SOC categories, the AEs associated with fluoxetine spanned all the SOC categories. AEs linked to escitalopram were observed across 15 SOC categories, whereas those related to sertraline were reported in 17 SOC categories. Notably, AEs for all three drugs were predominantly concentrated in three key SOC categories: psychiatric disorders; nervous system disorders; and injury, poisoning, and procedural complications. Of these, AEs associated with psychiatric disorders and nervous system disorders were particularly significant, prompting further HLGT-level analysis.

We conducted AE signal detection for fluoxetine, escitalopram, and sertraline in patients with psychiatric and neurological disorders via the ROR and PRR methods. In psychiatric disorders, fluoxetine, escitalopram, and sertraline had 1,523, 265, and 493 AE signals, respectively, involving 20 HLGTs. Fluoxetine was associated with 19 HLGTs, whereas escitalopram and sertraline each involved 14 HLGTs (Fig 2b). The most frequent AE signals were suicidal and self-injurious behaviors (NEC), followed by anxiety disorders and symptoms. In nervous system disorders, fluoxetine, escitalopram, and sertraline exhibited 941, 85, and 241 AE signals, respectively, covering 9 HLGTs. Fluoxetine and sertraline each involved 8 HLGTs, whereas escitalopram was associated with 5 HLGTs (Fig 2c). The most prominent AEs observed for these three drugs were neurological disorders (NEC) and motor disorders (including parkinsonism).

### Signal distribution and detection at the PT level

**Fig 3:**
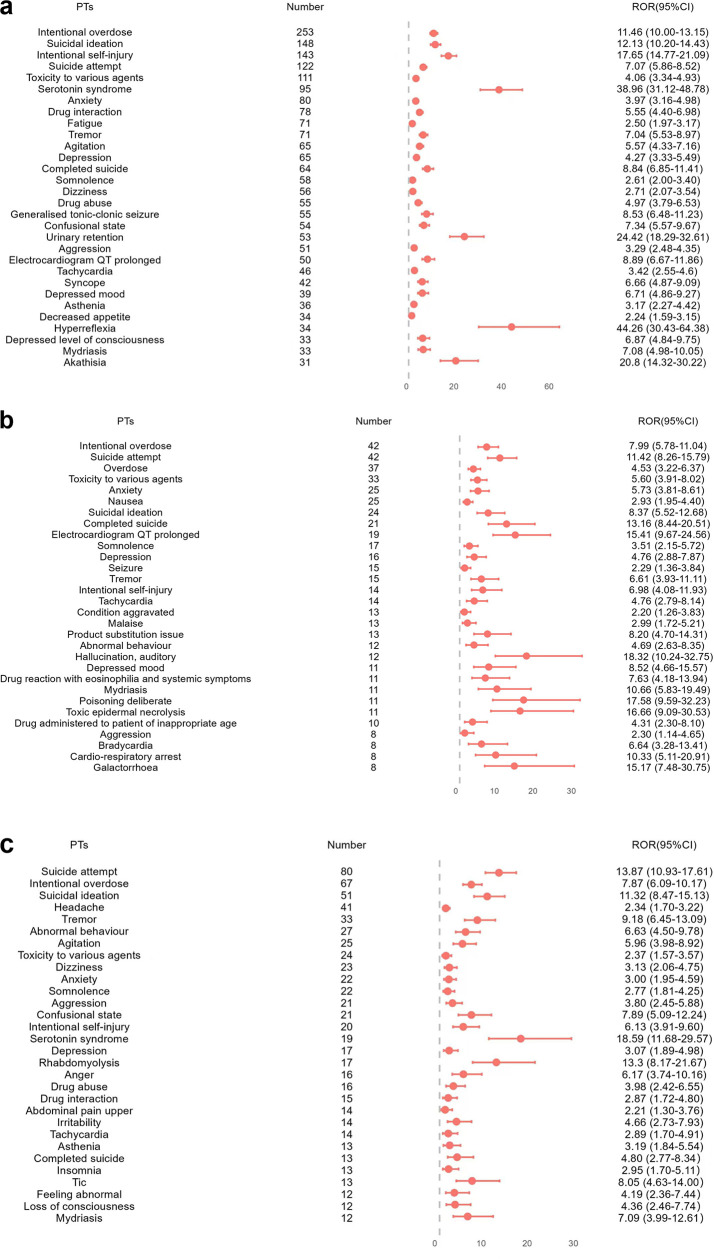
The top 30 AEs associated with (a)fluoxetine, (b)escitalopram, and (c)sertraline, respectively, ranked by the number of positive signals along with their PT and ROR values.

**Fig 4:**
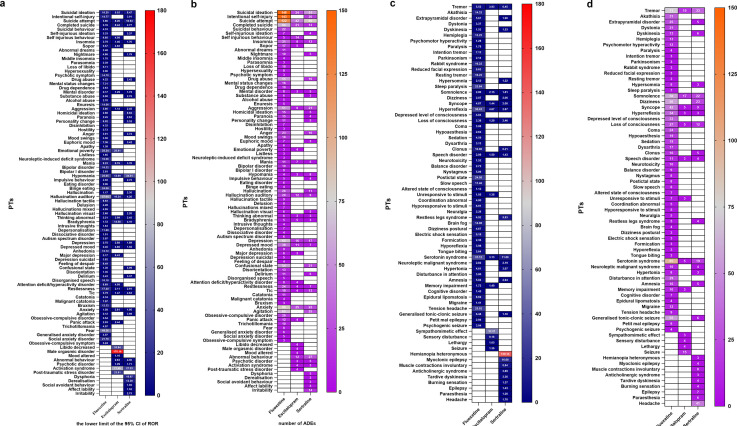
Distribution of PTs associated with psychiatric disorders (a, b) and nervous system disorders (c, d). In panels (a) and (c), the color gradient (blue to red) represents the lower limit of the 95% CI of ROR (range: 0–180). In panels (b) and (d), the gradient (purple to orange) corresponds to AE counts (range: 0–150).

After data filtering and sorting, fluoxetine was associated with 4,344 AEs across 299 PTs, escitalopram with 787 AEs across 104 PTs, and sertraline with 1,129 AEs across 118 PTs. The top 30 most frequently reported positive signals for each drug are presented in Figs 2–4.

Among the top 30 AEs with the highest signal frequencies, fluoxetine, escitalopram, and sertraline all demonstrated signals related to intentional overdose, suicidal ideation, suicide attempt, toxicity to various agents and anxiety. For fluoxetine, the top five reported signals were intentional overdose, suicidal ideation, intentional self-injury, suicide attempt, and toxicity to various agents, with stronger signals observed for decreased appetite, serotonin syndrome, urinary retention, and akathisia (Fig 3a). The top five signals of escitalopram included intentional overdose, suicide attempt, overdose, toxicity to various agents, and anxiety, with more pronounced associations for hallucination auditory, deliberate poisoning, toxic epidermal necrolysis, and QT prolongation (Fig 3b). The most common AEs associated with sertraline were suicide attempt, intentional overdose, suicidal ideation, headache, and tremor, with more prominent signals for serotonin syndrome, suicide attempt, rhabdomyolysis, and suicidal ideation (Fig 3c).

### Signal distribution and detection of PTs in psychiatric disorders

The distribution of PTs associated with psychiatric disorders induced by fluoxetine, escitalopram, and sertraline is illustrated in Fig 4a, with the corresponding absolute signal counts (without adjustment for prescription volume or patient exposure) depicted in Fig 4b. Fluoxetine showed the highest absolute number of signals, followed by sertraline, whereas escitalopram presented the lowest signal count. Specifically, for fluoxetine, the top five AE signals by report frequency included suicidal ideation, intentional self-injury, suicide attempt, anxiety, and depression. In terms of signal strength, the leading adverse events were auditory hallucinations, fear, hypomania, emotional poverty, and binge eating. For escitalopram, the most frequently reported adverse event signals were suicide attempt, anxiety, suicidal ideation, completed suicide, and depression. The top five adverse events by signal strength for escitalopram included male orgasmic disorder, activation syndrome, decreased libido, emotional poverty, and posttraumatic stress disorder. Regarding sertraline, the top five reported adverse event signals were suicide attempt, suicidal ideation, abnormal behavior, agitation, and anxiety. In terms of signal strength, the leading adverse events associated with sertraline were hypomania, activation syndrome, dysphoria, derealization, and suicide attempt.

### Signal distribution and detection of PTs in nervous system disorders

The intensity distribution of PTs in nervous system disorders associated with fluoxetine, escitalopram, and sertraline is shown in Fig 4c, whereas the absolute number of PT signals (without adjustment for prescription volume or patient exposure) for neurological conditions induced by these three drugs is shown in Fig 4d. Consistent with the distribution trend observed in nervous system disorders, fluoxetine results in the highest absolute number of signals, whereas escitalopram results in the lowest. Specifically, the top five adverse event signals reported for fluoxetine include serotonin syndrome, tremors, somnolence, dizziness, and generalized tonic‒clonic seizures. In terms of signal strength, the leading AEs were serotonin syndrome, hyperreflexia, tension headache, postictal state, and sleep paralysis. For escitalopram, the most frequently reported adverse event signals are somnolence, tremor, seizure, serotonin syndrome, and syncope. The top five adverse events by signal intensity for escitalopram included sympathomimetic effects, sensory disturbances, hyperreflexia, tremors, and serotonin syndrome. For sertraline, the top five reported AE signals were headache, tremor, dizziness, somnolence, and serotonin syndrome. In terms of signal intensity, the leading adverse events for sertraline were heteronymous hemianopia, myoclonic epilepsy, serotonin syndrome, restless leg syndrome, and involuntary muscle contractions.

## Discussion

Our study systematically evaluated the safety profiles of fluoxetine, escitalopram, and sertraline in children and adolescents aged 6–17 years using data from the FAERS between 2014 and 2023. Our analysis was conducted from three key dimensions: demographic characteristics, reporting patterns, and drug-specific AEs, aiming to provide evidence-based guidance for clinical practice. The following discussion will integrate existing evidence to focus on the safety characteristics of these SSRIs in pediatric populations and their clinical implications.

As presented in Table 3, the highest number of AEs was reported in adolescents aged 12–17 years, likely reflecting the greater antidepressant usage in this age group. Notably, the incidence of AEs in females was approximately twice that in males, a finding consistent with previous studies [31,32].Many studies have shown that gender differences were observed not only in AEs incidence but also in drug efficacy: SSRIs demonstrated better therapeutic effects in female patients [33]. This disparity may be attributed to a combination of physiological [34,35], pharmacokinetic [36], and neuroendocrine factors [37]. Females exhibit lower gastric acid secretion, higher body fat proportion, and reduced hepatic metabolic enzyme activity, leading to enhanced drug absorption, broader distribution, and slower metabolism, ultimately resulting in higher blood drug concentrations. From a neuroendocrine perspective, estrogen enhances antidepressant efficacy by upregulating serotonergic (5-HT) neurotransmission, while progesterone’s metabolite allopregnanolone (ALLO) synergistically potentiates GABAergic inhibition, combined with the female specific hypothalamic-pituitary-adrenal (HPA) axis hyperresponsiveness. The above aspects collectively constitute the foundation for gender differences in antidepressant efficacy. Furthermore, epidemiological studies indicate that the incidence of depression in women is significantly higher than in men, approximately twice as high as that in men [38–41], and this difference has emerged as early as adolescence. Consistently, Women were twice as likely as men to receive antidepressant prescription [42–44]. This pattern not only explains the higher rate of antidepressant prescriptions among women but also aligns with their increased reporting of AEs. Based on this evidence, we speculate that the observed gender differences in adverse events in this study may be largely attributable to the higher usage rate of antidepressant medications among female patients. Geographically, the United States reported the greatest number of AEs, followed by other Western nations such as the United Kingdom and France. This difference is mainly attributed to three factors: firstly, the United States has a larger population base; Secondly, its drug adverse reaction monitoring system is more comprehensive; Furthermore, there are regional differences in antidepressant prescribing patterns. This difference based on the absolute number of reports does not represent the true variations in the risk of AEs.

Notably, China emerged as the only Asian country among the top five, ranking second in sertraline-related AE reports. This prominence likely stems from the exclusive approval of sertraline for pediatric depression treatment in China. Furthermore, healthcare professionals constituted the majority of the AE reporters, reflecting both patient confidence in medical expertise and facilitating timely reporting of drug-related adverse reactions.

Our study analyzed AEs via the SOC and HLGT classifications, and revealed that fluoxetine, escitalopram, and sertraline have similar AE profiles, primarily with respect to psychiatric disorders and nervous system disorders.

The AEs associated with fluoxetine, the most common psychiatric disorder in children and adolescents, include suicidal ideation, intentional self-injury, and suicide attempts (all with >100 reported cases), followed by anxiety and agitation. While hallucinations, auditory disturbances, fear, hypomania, emotional blunting, and binge eating are strongly correlated, their occurrence rates remain relatively low. A FAERS-based study (2015–2019) identified similar high-frequency AEs, including serotonin syndrome and QT prolongation [27], which was consistent with our findings. Notably, the use of the fluoxetine package warns of increased suicide risk in patients <24 years old, supported by a network meta-analysis (OR 1.27, 95% CI 0.87–1.86) [7] and case reports dating back to 1990 [45,46], although the exact mechanism remains unclear. In nervous system disorders, fluoxetine is significantly associated with serotonin syndrome (ranking first in frequency and signal intensity) and extrapyramidal reactions (e.g., tremor, akathisia, and dystonia, occurring in approximately 20% of treated patients) [47–54]. Cardiovascular risks, particularly QT prolongation and associated torsade de pointes (TdP), are concerning despite low incidence, with mortality rates reaching 10–20% [55–57]. Recent analysis also highlighted the associations of fluoxetine with intentional overdose [58], decreased appetite, and insomnia [59,60]. Additionally, case reports confirmed the fluoxetine-induced urinary retention in pediatric patients, potentially leading to serious complications if untreated [61–64].

Similar to fluoxetine, suicidal behaviors remain prominent adverse events (AEs) of escitalopram, with suicide attempt ranking first and suicidal ideation third among psychiatric disorders [65]. While a meta-analysis suggested that escitalopram may slightly reduce suicide-related outcomes compared with placebo (OR 0.89, 95% CI 0.43–1.84) [7], the relationship between escitalopram and suicide risk remains controversial, necessitating large-scale, long-term studies with rigorous trial designs to establish clearer clinical guidelines. Among SSRIs, escitalopram had the strongest association with sexual dysfunction among SSRIs, particularly male orgasmic disorders (ROR 605.62, 95% CI 170.16–2155.52) and decreased libido (ROR 95.61, 95% CI 33.94–269.34). While these effects are typically reversible upon discontinuation, the use of fluoxetine or sertraline may be preferable for sexually active adolescents because of their lower risk of sexual AE risk [66–68]. In nervous system disorders, escitalopram is associated with significant cardiovascular concerns, with the strongest correlation with QT prolongation among pediatric antidepressants [69,70]. This is particularly relevant in overdose scenarios [71], despite meta-analyses suggesting better cardiovascular safety in elderly populations [55]. Additionally, escitalopram is uniquely associated with toxic epidermal necrolysis, a rare but severe cutaneous reaction with 14.8% mortality [72], although previous reports have focused primarily on fluoxetine and sertraline [73–75]. The high incidence of intentional overdose [76] underscores the need for strict medication adherence and professional supervision during escitalopram treatment.

Sertraline has a strong association with suicidal behaviors, ranking highest in both frequency and correlation among psychiatric disorder-related AEs. A network meta-analysis of 5260 pediatric patients with severe depression revealed that sertraline was associated with the highest suicide risk, followed by fluoxetine and escitalopram [10]. While another meta-analysis of 27 RCTs revealed increased suicidal ideation/attempt risk with antidepressants overall, the differences were not statistically significant across indications [77]. In nervous system disorders, common sertraline AEs include headache (incidence rate exceeding 10%), dizziness, tremor, somnolence, and serotonin syndrome. Although SSRIs could theoretically alleviate headaches through (Serotonin)5-HT modulation, clinical evidence does not support their efficacy over placebo for migraine prevention [78–80]. Serotonin syndrome, which occurs in approximately 14% of SSRI overdoses [81], requires early identification and prompt drug discontinuation, and is particularly challenging in pediatric patients because of variable presentations [82]. Notably, sertraline is associated with rhabdomyolysis, which typically emerges 1–4 months postinitiation or dose escalation, and is sometimes linked to overdose or exercise [83–87]. Although rare, this potentially life-threatening condition necessitates careful monitoring and early intervention. Like other SSRIs, intentional overdose remains a significant concern with sertraline, potentially leading to serotonin syndrome or chronic toxicity [82,88].

While previous studies have sporadically reported many of the AEs identified in this research, systematic investigations remain limited. Our findings not only confirm the AEs documented in the prescribing information for fluoxetine, escitalopram, and sertraline but also reveal numerous undocumented adverse reactions. The in-depth analysis of these AEs provides clinicians with valuable guidance for managing similar cases in practice.

## Conclusions

Our study compared the adverse effects of fluoxetine, escitalopram, and sertraline in pediatric populations. All three medications demonstrated similar high-risk adverse reactions (including suicidal ideation, self-harm behaviors, and drug overdose), while each exhibited unique risks: fluoxetine was associated with serotonin syndrome, escitalopram was prone to cause QT prolongation, severe skin reactions, and sexual dysfunction, and sertraline more frequently led to headaches and rhabdomyolysis. Additionally, we identified several previously undocumented adverse reactions in drug labeling information, such as disorientation and trichotillomania with fluoxetine, syncope and alopecia with escitalopram, and amnesia and photosensitivity with sertraline. To the best of our knowledge, this represents the largest comparative study to date on the safety profiles of antidepressants in children, providing crucial evidence for clinical medication selection. The findings highlight the need for physicians to exercise particular caution with high-dose regimens, long-term use, or combination therapies, while emphasizing the importance of ongoing pharmacovigilance to improve drug safety information.

## Supporting information

S1 Table Fourfold table for disproportionality analyses. (DOCX)

S2 Table Computational formula and threshold of signal detection. (DOCX)

S3 Table Basic information of AE reports. (DOCX)

S4 Table The top 30 AEs associated with fluoxetine, ranked by the number of positive signals along with their PT and ROR values. (DOCX)

S5 Table The top 30 AEs associated with escitalopram, ranked by the number of positive signals along with their PT and ROR values. (DOCX)

S6 Table The top 30 AEs associated with sertraline, ranked by the number of positive signals along with their PT and ROR values. (DOCX)

S7 Table Distribution of PTs for psychiatric disorders along with their the lower limit of the 95%CI of ROR. (DOCX)

S8 Table Distribution of PTs for nervous system disorders along with their the lower limit of the 95%CI of ROR. (DOCX)

S9 Table Distribution of PTs for psychiatric disorders along with their number of ADEs. (DOCX)

S10 Table Distribution of PTs for nervous system disorders along with their number of ADEs. (DOCX)

S11 Table AE distributions for fluoxetine, escitalopram, and sertraline at the SOC level. (DOCX)

S12 Table HLGT distribution of psychiatric disorders event signals. (DOCX)

S13 Table HLGT distribution of nervous system disorders event signals. (DOCX)
