# Supplementary material for: Safety of antidepressants commonly used in 6–17-year-old children and adolescents: A disproportionality analysis from 2014–2023 on the basis of the FAERS database
Source: PLoS One. 2025 Aug 13;20(8):e0330025. doi: 10.1371/journal.pone.0330025 (PMC12349705; doi:10.1371/journal.pone.0330025)
Supplement: S1 Table — (DOCX) [file pone.0330025.s001.docx]

**S1 Table. Fourfold table for disproportionality analyses.**

| **Event groups** | **Drug used** | **Other drugs** | **Sums** |
| --- | --- | --- | --- |
| Event | a | c | a+c |
| Other events | b | d | b+d |
| Sums | a+b | c+d | a+b+c+d |
