# Supplementary material for: Safety of antidepressants commonly used in 6–17-year-old children and adolescents: A disproportionality analysis from 2014–2023 on the basis of the FAERS database
Source: PLoS One. 2025 Aug 13;20(8):e0330025. doi: 10.1371/journal.pone.0330025 (PMC12349705; doi:10.1371/journal.pone.0330025)
Supplement: S2 Table — (DOCX) [file pone.0330025.s002.docx]

**S2 Table. Computational formula and threshold of signal detection.**

| **Algorithms** | **Equation** | **Criteria** |
| --- | --- | --- |
| ROR | ROR =ad/bc | 95%CI > 1, N ≥ 3 |
|  | 95%CI=e^ln(ROR)±1.96(1/a+1/b+1/c+1/d)^0.5^ |  |
| PRR | PRR =a(c + d)/c/(a + b) | PRR≥2, χ^2^ ≥ 4, N ≥ 3 |
|  | χ^2^=[(ad−bc)^2](a + b + c + d)/[(a + b)(c +d)(a + c)(b + d)] |  |
