# Supplementary material for: Safety of antidepressants commonly used in 6–17-year-old children and adolescents: A disproportionality analysis from 2014–2023 on the basis of the FAERS database
Source: PLoS One. 2025 Aug 13;20(8):e0330025. doi: 10.1371/journal.pone.0330025 (PMC12349705; doi:10.1371/journal.pone.0330025)
Supplement: S3 Table — (DOCX) [file pone.0330025.s003.docx]

**S3 Table. Basic information of AE reports.**

| **Characteristics** | **Fluoxetine(N=1604)** | **Escitalopram(N=352)** | **Sertraline(N=571)** |
| --- | --- | --- | --- |
| ***Age (years)*** |  |  |  |
| Mean(SD) | 14.47(2.38) | 14.78(2.37) | 14.01(2.65) |
| Median(Q1,Q3) | 15.00(14-16) | 15.00(14-17) | 15.00(13-16) |
| 6-8 | 54(3.37) | 13(3.69) | 28(4.90) |
| 9-11 | 127(7.92) | 19(5.40) | 64(11.21) |
| 12-15 | 796(49.63) | 154(43.75) | 284(49.74) |
| 16-17 | 627(39.09) | 166(47.16) | 195(34.15) |
| ***Gender (n,%)*** |  |  |  |
| Male | 522(32.54) | 96(29.54) | 191(33.45) |
| Female | 993(61.91) | 238(73.23) | 335(58.67) |
| Unknown | 89(5.55) | 18(5.54) | 45(7.88) |
| ***Type of reporter*** |  |  |  |
| Health professional | 1089(67.89) | 246(69.89) | 318(55.69) |
| Non health professional | 475(29.61) | 86(24.43) | 237(41.51) |
| Unknown | 40(2.49) | 20(5.68) | 16(2.80) |
| ***Reporting countries (Top five)*** |  |  |  |
|  | US 553(34.48) | US 192(54.55) | US 255(44.66) |
|  | GB 276(17.21) | FR 28(7.95) | CN 55(9.63) |
|  | FR 139(8.67) | DE 22(6.25) | FR 36(6.30) |
|  | SE 97(6.05) | DK 19(5.40) | HR 36(6.30) |
|  | DE 94(5.86) | CA 15(4.26) | IT 32(5.60) |
