# Supplementary material for: Safety of antidepressants commonly used in 6–17-year-old children and adolescents: A disproportionality analysis from 2014–2023 on the basis of the FAERS database
Source: PLoS One. 2025 Aug 13;20(8):e0330025. doi: 10.1371/journal.pone.0330025 (PMC12349705; doi:10.1371/journal.pone.0330025)
Supplement: S4 Table — (DOCX) [file pone.0330025.s004.docx]

**S4 Table. The top 30 AEs associated with fluoxetine, ranked by the number of positive signals along with their PT and ROR values.**

| **PT(Preferred Terms)** | **N** | **ROR(95%Cl)** |
| --- | --- | --- |
| Intentional overdose | 253 | 11.46(10.00-13.15) |
| Suicidal ideation | 148 | 12.13(10.20-14.43) |
| Intentional self-injury | 143 | 17.65(14.77-21.09) |
| Suicide attempt | 122 | 7.07(5.86-8.52) |
| Toxicity to various agents | 111 | 4.06(3.34-4.93) |
| Serotonin syndrome | 95 | 38.96(31.12-48.78) |
| Anxiety | 80 | 3.97(3.16-4.98) |
| Drug interaction | 78 | 5.55(4.40-6.98) |
| Fatigue | 71 | 2.50(1.97-3.17) |
| Tremor | 71 | 7.04(5.53-8.97) |
| Agitation | 65 | 5.57(4.33-7.16) |
| Depression | 65 | 4.27(3.33-5.49) |
| Completed suicide | 64 | 8.84(6.85-11.41) |
| Somnolence | 58 | 2.61(2.00-3.40) |
| Dizziness | 56 | 2.71(2.07-3.54) |
| Drug abuse | 55 | 4.97(3.79-6.53) |
| Generalised tonic-clonic seizure | 55 | 8.53(6.48-11.23) |
| Confusional state | 54 | 7.34(5.57-9.67) |
| Urinary retention | 53 | 24.42(18.29-32.61) |
| Aggression | 51 | 3.29(2.48-4.35) |
| Electrocardiogram QT prolonged | 50 | 8.89(6.67-11.86) |
| Tachycardia | 46 | 3.42(2.55-4.6) |
| Syncope | 42 | 6.66(4.87-9.09) |
| Depressed mood | 39 | 6.71(4.86-9.27) |
| Asthenia | 36 | 3.17(2.27-4.42) |
| Decreased appetite | 34 | 2.24(1.59-3.15) |
| Hyperreflexia | 34 | 44.26(30.43-64.38) |
| Depressed level of consciousness | 33 | 6.87(4.84-9.75) |
| Mydriasis | 33 | 7.08（4.98-10.05) |
| Akathisia | 31 | 20.8（14.32-30.22) |
