# Supplementary material for: Safety of antidepressants commonly used in 6–17-year-old children and adolescents: A disproportionality analysis from 2014–2023 on the basis of the FAERS database
Source: PLoS One. 2025 Aug 13;20(8):e0330025. doi: 10.1371/journal.pone.0330025 (PMC12349705; doi:10.1371/journal.pone.0330025)
Supplement: S5 Table — (DOCX) [file pone.0330025.s005.docx]

**S5 Table. The top 30 AEs associated with escitalopram, ranked by the number of positive signals along with their PT and ROR values.**

| **PT(Preferred Terms)** | **N** | **ROR(95%Cl)** |
| --- | --- | --- |
| Intentional overdose | 42 | 7.99(5.78-11.04) |
| Suicide attempt | 42 | 11.42(8.26-15.79) |
| Overdose | 37 | 4.53(3.22-6.37) |
| Toxicity to various agents | 33 | 5.60(3.91-8.02) |
| Anxiety | 25 | 5.73(3.81-8.61) |
| Nausea | 25 | 2.93(1.95-4.40) |
| Suicidal ideation | 24 | 8.37(5.52-12.68) |
| Completed suicide | 21 | 13.16(8.44-20.51) |
| Electrocardiogram QT prolonged | 19 | 15.41(9.67-24.56) |
| Somnolence | 17 | 3.51(2.15-5.72) |
| Depression | 16 | 4.76(2.88-7.87) |
| Seizure | 15 | 2.29(1.36-3.84) |
| Tremor | 15 | 6.61(3.93-11.11) |
| Intentional self-injury | 14 | 6.98(4.08-11.93) |
| Tachycardia | 14 | 4.76(2.79-8.14) |
| Condition aggravated | 13 | 2.20(1.26-3.83) |
| Malaise | 13 | 2.99(1.72-5.21) |
| Product substitution issue | 13 | 8.20(4.70-14.31) |
| Abnormal behaviour | 12 | 4.69(2.63-8.35) |
| Hallucination, auditory | 12 | 18.32(10.24-32.75) |
| Depressed mood | 11 | 8.52(4.66-15.57) |
| Drug reaction with eosinophilia and systemic symptoms | 11 | 7.63(4.18-13.94) |
| Mydriasis | 11 | 10.66(5.83-19.49) |
| Poisoning deliberate | 11 | 17.58(9.59-32.23) |
| Toxic epidermal necrolysis | 11 | 16.66(9.09-30.53) |
| Drug administered to patient of inappropriate age | 10 | 4.31(2.30-8.10) |
| Aggression | 8 | 2.30(1.14-4.65) |
| Bradycardia | 8 | 6.64(3.28-13.41) |
| Cardio-respiratory arrest | 8 | 10.33(5.11-20.91) |
| Galactorrhoea | 8 | 15.17(7.48-30.75) |
