# Supplementary material for: Safety of antidepressants commonly used in 6–17-year-old children and adolescents: A disproportionality analysis from 2014–2023 on the basis of the FAERS database
Source: PLoS One. 2025 Aug 13;20(8):e0330025. doi: 10.1371/journal.pone.0330025 (PMC12349705; doi:10.1371/journal.pone.0330025)
Supplement: S6 Table — (DOCX) [file pone.0330025.s006.docx]

**S6 Table. The top 30 AEs associated with sertraline, ranked by the number of positive signals along with their PT and ROR values.**

| **PT(Preferred Terms)** | **N** | **ROR(95%Cl)** |
| --- | --- | --- |
| Suicide attempt | 80 | 13.87(10.93-17.61) |
| Intentional overdose | 67 | 7.87(6.09-10.17) |
| Suicidal ideation | 51 | 11.32(8.47-15.13) |
| Headache | 41 | 2.34(1.70-3.22) |
| Tremor | 33 | 9.18(6.45-13.09) |
| Abnormal behaviour | 27 | 6.63(4.50-9.78) |
| Agitation | 25 | 5.96(3.98-8.92) |
| Toxicity to various agents | 24 | 2.37(1.57-3.57) |
| Dizziness | 23 | 3.13(2.06-4.75) |
| Anxiety | 22 | 3.00(1.95-4.59) |
| Somnolence | 22 | 2.77(1.81-4.25) |
| Aggression | 21 | 3.80(2.45-5.88) |
| Confusional state | 21 | 7.89(5.09-12.24) |
| Intentional self-injury | 20 | 6.13(3.91-9.60) |
| Serotonin syndrome | 19 | 18.59(11.68-29.57) |
| Depression | 17 | 3.07(1.89-4.98) |
| Rhabdomyolysis | 17 | 13.3(8.17-21.67) |
| Anger | 16 | 6.17(3.74-10.16) |
| Drug abuse | 16 | 3.98(2.42-6.55) |
| Drug interaction | 15 | 2.87(1.72-4.80) |
| Abdominal pain upper | 14 | 2.21(1.30-3.76) |
| Irritability | 14 | 4.66(2.73-7.93) |
| Tachycardia | 14 | 2.89(1.70-4.91) |
| Asthenia | 13 | 3.19(1.84-5.54) |
| Completed suicide | 13 | 4.80(2.77-8.34） |
| Insomnia | 13 | 2.95(1.70-5.11） |
| Tic | 13 | 8.05(4.63-14.00） |
| Feeling abnormal | 12 | 4.19(2.36-7.44） |
| Loss of consciousness | 12 | 4.36(2.46-7.74） |
| Mydriasis | 12 | 7.09(3.99-12.61） |
