# Supplementary material for: Safety of antidepressants commonly used in 6–17-year-old children and adolescents: A disproportionality analysis from 2014–2023 on the basis of the FAERS database
Source: PLoS One. 2025 Aug 13;20(8):e0330025. doi: 10.1371/journal.pone.0330025 (PMC12349705; doi:10.1371/journal.pone.0330025)
Supplement: S7 Table — (DOCX) [file pone.0330025.s007.docx]

**S7 Table. Distribution of PTs for psychiatric disorders along with their the lower limit of the 95%CI of ROR.**

| **PT(Preferred Terms)** | **Fluoxetine** | **Escitalopram** | **Sertraline** |
| --- | --- | --- | --- |
| Suicidal ideation | 10.20 | 5.52 | 8.47 |
| Intentional self-injury | 14.77 |  | 3.91 |
| Suicide attempt | 5.86 | 8.26 | 10.93 |
| Completed suicide | 3.16 | 8.44 | 2.77 |
| Suicidal behaviour | 4.33 |  |  |
| Self-injurious ideation | 3.33 |  | 3.37 |
| Self injurious behaviour | 6.85 | 4.08 |  |
| Insomnia | 3.79 | 1.46 | 1.70 |
| Sopor | 5.57 | 4.82 |  |
| Abnormal dreams | 2.48 |  |  |
| Nightmare | 4.86 |  | 1.79 |
| Middle insomnia | 1.75 |  |  |
| Parasomnia | 2.39 |  |  |
| Loss of libido | 5.11 |  |  |
| Hypersexuality | 2.06 |  |  |
| Psychotic symptom | 14.70 |  |  |
| Drug abuse | 4.22 |  | 2.42 |
| Mental status changes | 7.31 |  |  |
| Drug dependence | 2.63 |  |  |
| Mental disorder | 2.17 | 1.29 | 1.72 |
| Substance abuse | 5.50 |  | 2.75 |
| Alcohol abuse | 2.70 |  |  |
| Enuresis | 14.56 |  |  |
| Aggression | 5.80 | 1.14 | 2.45 |
| Homicidal ideation | 2.88 |  | 8.82 |
| Paranoia | 2.45 |  | 2.63 |
| Personality change | 9.02 |  | 3.32 |
| Disinhibition | 4.32 |  |  |
| Hostility | 2.73 |  |  |
| Anger | 3.91 |  | 3.74 |
| Mood swings | 3.50 |  |  |
| Euphoric mood | 7.36 |  | 3.42 |
| Apathy | 1.38 |  |  |
| Emotional poverty | 25.24 | 25.61 |  |
| Listless | 1.65 |  |  |
| Neuroleptic-induced deficit syndrome | 16.06 |  |  |
| Mania | 9.23 | 9.76 | 3.70 |
| Bipolar disorder | 16.22 |  |  |
| Bipolar I disorder | 2.93 |  |  |
| Hypomania | 25.65 | 12.69 | 34.51 |
| Impulsive behaviour | 4.02 |  | 2.75 |
| Eating disorder | 2.06 |  |  |
| Binge eating | 24.08 |  |  |
| Hallucination | 1.17 |  | 2.30 |
| Hallucination auditory | 57.04 | 10.24 | 4.26 |
| Hallucination tactile | 8.93 |  |  |
| Delusion | 4.28 |  |  |
| Hallucinations mixed | 4.95 |  |  |
| Hallucination visual | 2.68 |  | 2.20 |
| Thinking abnormal | 2.21 | 3.99 | 2.45 |
| Bradyphrenia | 1.72 | 14.58 | 4.10 |
| Intrusive thoughts | 1.63 |  |  |
| Depersonalisation | 11.68 |  |  |
| Dissociative disorder | 1.01 |  |  |
| Autism spectrum disorder | 3.97 |  |  |
| Depression | 2.75 | 2.88 | 1.89 |
| Depressed mood | 2.45 | 4.66 | 1.55 |
| Anhedonia | 12.72 |  |  |
| Major depression | 3.17 | 6.43 |  |
| Depression suicidal | 3.82 |  |  |
| Feeling of despair | 8.49 |  |  |
| Confusional state | 1.30 |  | 5.09 |
| Disorientation | 1.35 |  |  |
| Delirium | 3.43 |  | 3.37 |
| Disorganised speech | 10.18 |  |  |
| Attention deficit/hyperactivity disorder | 8.69 | 4.98 |  |
| Restlessness | 17.40 | 2.11 | 2.91 |
| Tic | 5.79 | 1.47 | 4.63 |
| Catatonia | 4.34 |  |  |
| Malignant catatonia | 7.57 |  |  |
| Bruxism | 12.32 |  |  |
| Anxiety | 1.58 | 3.81 | 1.95 |
| Agitation | 2.12 |  | 3.98 |
| Obsessive-compulsive disorder | 3.54 |  |  |
| Panic attack | 2.03 | 2.44 |  |
| Trichotillomania | 4.07 |  |  |
| Fear | 49.26 |  |  |
| Generalised anxiety disorder | 4.07 |  |  |
| Social anxiety disorder | 21.78 |  |  |
| Obsessive-compulsive symptom | 3.24 |  |  |
| Libido decreased |  | 33.94 |  |
| Male orgasmic disorder |  | 170.16 |  |
| Mood altered |  | 1.10 |  |
| Abnormal behaviour |  | 2.63 | 4.50 |
| Psychotic disorder |  | 1.29 | 1.71 |
| Activation syndrome |  | 71.56 | 27.23 |
| Post-traumatic stress disorder |  | 23.81 |  |
| Dysphoria |  |  | 16.97 |
| Derealisation |  |  | 12.28 |
| Social avoidant behaviour |  |  | 1.92 |
| Affect lability |  |  | 1.52 |
| Irritability |  |  | 2.73 |
