# Supplementary material for: Safety of antidepressants commonly used in 6–17-year-old children and adolescents: A disproportionality analysis from 2014–2023 on the basis of the FAERS database
Source: PLoS One. 2025 Aug 13;20(8):e0330025. doi: 10.1371/journal.pone.0330025 (PMC12349705; doi:10.1371/journal.pone.0330025)
Supplement: S8 Table — (DOCX) [file pone.0330025.s008.docx]

**S8 Table. Distribution of PTs for nervous system disorders along with their the lower limit of the 95%CI of ROR.**

| **PT(Preferred Terms)** | **Fluoxetine** | **Escitalopram** | **Sertraline** |
| --- | --- | --- | --- |

| Tremor | 5.53 | 3.93 | 6.45 |
| --- | --- | --- | --- |
| Akathisia | 14.32 |  |  |
| Extrapyramidal disorder | 5.67 |  | 1.90 |
| Dystonia | 3.37 |  |  |
| Dyskinesia | 1.53 |  | 1.53 |
| Hemiplegia | 13.25 |  |  |
| Psychomotor hyperactivity | 1.28 |  |  |
| Paralysis | 1.78 |  |  |
| Intention tremor | 7.12 |  |  |
| Parkinsonism | 2.14 |  |  |
| Rabbit syndrome | 16.22 |  |  |
| Reduced facial expression | 9.65 |  |  |
| Resting tremor | 16.22 |  |  |
| Hypersomnia | 2.12 |  | 1.22 |
| Sleep paralysis | 23.84 |  |  |
| Somnolence | 2.00 | 2.15 | 1.81 |
| Dizziness | 2.07 |  | 2.06 |
| Syncope | 4.87 | 1.44 | 2.00 |
| Hyperreflexia | 30.43 | 4.67 | 2.87 |
| Depressed level of consciousness | 4.84 |  |  |
| Loss of consciousness | 2.38 | 1.20 | 2.46 |
| Coma | 3.08 |  |  |
| Hypoaesthesia | 2.62 |  |  |
| Sedation | 4.59 |  |  |
| Dysarthria | 3.13 |  |  |
| Clonus | 10.96 |  | 6.21 |
| Speech disorder | 2.09 | 1.50 | 1.43 |
| Neurotoxicity | 1.52 |  |  |
| Balance disorder | 2.72 |  |  |
| Nystagmus | 5.09 |  |  |
| Postictal state | 24.08 |  |  |
| Slow speech | 11.46 |  |  |
| Altered state of consciousness | 1.12 |  |  |
| Unresponsive to stimuli | 1.05 | 1.38 |  |
| Coordination abnormal | 4.00 |  |  |
| Hyporesponsive to stimuli | 6.91 |  |  |
| Neuralgia | 2.38 |  |  |
| Restless legs syndrome | 4.92 |  | 9.93 |
| Brain fog | 14.08 |  |  |
| Dizziness postural | 4.67 |  |  |
| Electric shock sensation | 9.18 |  |  |
| Formication | 2.36 |  |  |
| Hyporeflexia | 3.98 |  |  |
| Tongue biting | 5.64 |  |  |
| Serotonin syndrome | 31.12 | 3.13 | 11.68 |
| Neuroleptic malignant syndrome | 2.82 |  | 2.19 |
| Hypertonia | 4.61 |  | 3.07 |
| Disturbance in attention | 3.00 |  |  |
| Amnesia | 5.04 |  | 2.94 |
| Memory impairment | 1.72 | 1.40 |  |
| Cognitive disorder | 1.45 |  |  |
| Epidural lipomatosis | 8.69 |  |  |
| Migraine | 1.41 |  |  |
| Tension headache | 25.02 |  |  |
| Generalised tonic-clonic seizure | 6.48 |  | 1.10 |
| Petit mal epilepsy | 2.94 |  |  |
| Psychogenic seizure | 3.04 |  |  |
| Sympathomimetic effect |  | 52.21 |  |
| Sensory disturbance |  | 9.16 |  |
| Lethargy |  | 1.05 |  |
| Seizure |  | 1.36 |  |
| Hemianopia heteronymous |  |  | 139.14 |
| Myoclonic epilepsy |  |  | 16.68 |
| Muscle contractions involuntary |  |  | 8.84 |
| Anticholinergic syndrome |  |  | 3.89 |
| Tardive dyskinesia |  |  | 3.36 |
| Burning sensation |  |  | 1.37 |
| Epilepsy |  |  | 1.43 |
| Paraesthesia |  |  | 1.20 |
| Headache |  |  | 1.70 |
