# Supplementary material for: Safety of antidepressants commonly used in 6–17-year-old children and adolescents: A disproportionality analysis from 2014–2023 on the basis of the FAERS database
Source: PLoS One. 2025 Aug 13;20(8):e0330025. doi: 10.1371/journal.pone.0330025 (PMC12349705; doi:10.1371/journal.pone.0330025)
Supplement: S9 Table — (DOCX) [file pone.0330025.s009.docx]

**S9 Table. Distribution of PTs for psychiatric disorders along with their number of ADEs.**

| **PT(Preferred Terms)** | **Fluoxetine** | **Escitalopram** | **Sertraline** |
| --- | --- | --- | --- |
| Suicidal ideation | 148 | 24 | 51 |
| Intentional self-injury | 143 |  | 20 |
| Suicide attempt | 122 | 42 | 80 |
| Completed suicide | 64 | 21 | 13 |
| Suicidal behaviour | 22 |  |  |
| Self-injurious ideation | 7 |  | 4 |
| Self injurious behaviour | 5 | 14 |  |
| Insomnia | 31 | 8 | 13 |
| Sopor | 17 | 5 |  |
| Abnormal dreams | 11 |  |  |
| Nightmare | 10 |  | 5 |
| Middle insomnia | 4 |  |  |
| Parasomnia | 3 |  |  |
| Loss of libido | 10 |  |  |
| Hypersexuality | 8 |  |  |
| Psychotic symptom | 3 |  |  |
| Drug abuse | 55 |  | 16 |
| Mental status changes | 16 |  |  |
| Drug dependence | 10 |  |  |
| Mental disorder | 8 | 3 | 5 |
| Substance abuse | 6 |  | 3 |
| Alcohol abuse | 5 |  |  |
| Enuresis | 5 |  |  |
| Aggression | 51 | 8 | 21 |
| Homicidal ideation | 15 |  | 5 |
| Paranoia | 15 |  | 4 |
| Personality change | 11 |  | 5 |
| Disinhibition | 7 |  |  |
| Hostility | 3 |  |  |
| Anger | 23 |  | 16 |
| Mood swings | 18 |  |  |
| Euphoric mood | 7 |  | 3 |
| Apathy | 6 |  |  |
| Emotional poverty | 3 | 4 |  |
| Listless | 3 |  |  |
| Neuroleptic-induced deficit syndrome | 3 |  |  |
| Mania | 11 | 7 | 5 |
| Bipolar disorder | 10 |  |  |
| Bipolar I disorder | 8 |  |  |
| Hypomania | 4 | 3 | 8 |
| Impulsive behaviour | 5 |  | 4 |
| Eating disorder | 6 |  |  |
| Binge eating | 4 |  |  |
| Hallucination | 26 |  | 11 |
| Hallucination auditory | 20 | 12 | 9 |
| Hallucination tactile | 9 |  |  |
| Delusion | 8 |  |  |
| Hallucinations mixed | 7 |  |  |
| Hallucination visual | 6 |  | 5 |
| Thinking abnormal | 6 | 3 | 3 |
| Bradyphrenia | 5 | 5 | 3 |
| Intrusive thoughts | 4 |  |  |
| Depersonalisation | 7 |  |  |
| Dissociative disorder | 4 |  |  |
| Autism spectrum disorder | 6 |  |  |
| Depression | 65 | 16 | 17 |
| Depressed mood | 39 | 11 | 7 |
| Anhedonia | 9 |  |  |
| Major depression | 6 | 3 |  |
| Depression suicidal | 5 |  |  |
| Feeling of despair | 4 |  |  |
| Confusional state | 54 |  | 21 |
| Disorientation | 13 |  |  |
| Delirium | 11 |  | 6 |
| Disorganised speech | 5 |  |  |
| Attention deficit/hyperactivity disorder | 8 | 4 |  |
| Restlessness | 24 | 4 | 7 |
| Tic | 19 | 4 | 13 |
| Catatonia | 11 |  |  |
| Malignant catatonia | 4 |  |  |
| Bruxism | 3 |  |  |
| Anxiety | 80 | 25 | 22 |
| Agitation | 65 |  | 25 |
| Obsessive-compulsive disorder | 20 |  |  |
| Panic attack | 12 | 4 |  |
| Trichotillomania | 10 |  |  |
| Fear | 8 |  |  |
| Generalised anxiety disorder | 6 |  |  |
| Social anxiety disorder | 5 |  |  |
| Obsessive-compulsive symptom | 3 |  |  |
| Libido decreased |  | 4 |  |
| Male orgasmic disorder |  | 4 |  |
| Mood altered |  | 4 |  |
| Abnormal behaviour |  | 12 | 27 |
| Psychotic disorder |  | 3 | 5 |
| Activation syndrome |  | 4 | 3 |
| Post-traumatic stress disorder |  | 4 |  |
| Dysphoria |  |  | 5 |
| Derealisation |  |  | 3 |
| Social avoidant behaviour |  |  | 3 |
| Affect lability |  |  | 3 |
| Irritability |  |  | 14 |
