# Supplementary material for: Safety of antidepressants commonly used in 6–17-year-old children and adolescents: A disproportionality analysis from 2014–2023 on the basis of the FAERS database
Source: PLoS One. 2025 Aug 13;20(8):e0330025. doi: 10.1371/journal.pone.0330025 (PMC12349705; doi:10.1371/journal.pone.0330025)
Supplement: S10 Table — (DOCX) [file pone.0330025.s010.docx]

**S10 Table. Distribution of PTs for nervous system disorders along with their number of ADEs.**

| **PT(Preferred Terms)** | **Fluoxetine** | **Escitalopram** | | **Sertraline** |
| --- | --- | --- | --- | --- |
| Tremor | 71 | | 15 | 33 |
| Akathisia | 31 | |  |  |
| Extrapyramidal disorder | 25 | |  | 5 |
| Dystonia | 21 | |  |  |
| Dyskinesia | 13 | |  | 6 |
| Hemiplegia | 13 | |  |  |
| Psychomotor hyperactivity | 12 | |  |  |
| Paralysis | 4 | |  |  |
| Intention tremor | 3 | |  |  |
| Parkinsonism | 3 | |  |  |
| Rabbit syndrome | 3 | |  |  |
| Reduced facial expression | 3 | |  |  |
| Resting tremor | 3 | |  |  |
| Hypersomnia | 9 | |  | 3 |
| Sleep paralysis | 7 | |  |  |
| Somnolence | 58 | | 17 | 22 |
| Dizziness | 56 | |  | 23 |
| Syncope | 42 | | 5 | 9 |
| Hyperreflexia | 34 | | 3 | 3 |
| Depressed level of consciousness | 33 | |  |  |
| Loss of consciousness | 27 | | 5 | 12 |
| Coma | 24 | |  |  |
| Hypoaesthesia | 19 | |  |  |
| Sedation | 19 | |  |  |
| Dysarthria | 17 | |  |  |
| Clonus | 16 | |  | 5 |
| Speech disorder | 11 | | 3 | 4 |
| Neurotoxicity | 10 | |  |  |
| Balance disorder | 9 | |  |  |
| Nystagmus | 8 | |  |  |
| Postictal state | 8 | |  |  |
| Slow speech | 8 | |  |  |
| Altered state of consciousness | 7 | |  |  |
| Unresponsive to stimuli | 7 | | 3 |  |
| Coordination abnormal | 5 | |  |  |
| Hyporesponsive to stimuli | 5 | |  |  |
| Neuralgia | 5 | |  |  |
| Restless legs syndrome | 5 | |  | 4 |
| Brain fog | 4 | |  |  |
| Dizziness postural | 3 | |  |  |
| Electric shock sensation | 3 | |  |  |
| Formication | 3 | |  |  |
| Hyporeflexia | 3 | |  |  |
| Tongue biting | 3 | |  |  |
| Serotonin syndrome | 95 | | 5 | 19 |
| Neuroleptic malignant syndrome | 10 | |  | 4 |
| Hypertonia | 8 | |  | 3 |
| Disturbance in attention | 29 | |  |  |
| Amnesia | 16 | |  | 5 |
| Memory impairment | 10 | | 3 |  |
| Cognitive disorder | 7 | |  |  |
| Epidural lipomatosis | 4 | |  |  |
| Migraine | 12 | |  |  |
| Tension headache | 9 | |  |  |
| Generalised tonic-clonic seizure | 55 | |  | 6 |
| Petit mal epilepsy | 9 | |  |  |
| Psychogenic seizure | 4 | |  |  |
| Sympathomimetic effect |  | | 3 |  |
| Sensory disturbance |  | | 4 |  |
| Lethargy |  | | 4 |  |
| Seizure |  | | 15 |  |
| Hemianopia heteronymous |  | |  | 3 |
| Myoclonic epilepsy |  | |  | 4 |
| Muscle contractions involuntary |  | |  | 8 |
| Anticholinergic syndrome |  | |  | 3 |
| Tardive dyskinesia |  | |  | 4 |
| Burning sensation |  | |  | 4 |
| Epilepsy |  | |  | 7 |
| Paraesthesia |  | |  | 6 |
| Headache |  | |  | 41 |
