# Supplementary material for: Safety of antidepressants commonly used in 6–17-year-old children and adolescents: A disproportionality analysis from 2014–2023 on the basis of the FAERS database
Source: PLoS One. 2025 Aug 13;20(8):e0330025. doi: 10.1371/journal.pone.0330025 (PMC12349705; doi:10.1371/journal.pone.0330025)
Supplement: S11 Table — (DOCX) [file pone.0330025.s011.docx]

**S11 Table. AE distributions for fluoxetine, escitalopram, and sertraline at the SOC level.**

| **SOC(System Organ Class)** | **Fluoxetine**  **(%)** | **Escitalopram**  **(%)** | **Sertraline**  **(%)** |
| --- | --- | --- | --- |
| Psychiatric disorders | 35.22 | 34.91 | 43.82 |
| Nervous system disorders | 21.76 | 11.20 | 21.42 |
| Injury, poisoning and procedural complications | 10.11 | 19.37 | 9.33 |
| General disorders and administration site conditions | 7.17 | 5.93 | 6.49 |
| Investigations | 5.02 | 4.48 | 1.24 |
| Cardiac disorders | 3.63 | 5.80 | 3.11 |
| Eye disorders | 3.15 | 2.11 | 2.31 |
| Musculoskeletal and connective tissue disorders | 2.66 | 1.32 | 4.00 |
| Hepatobiliary disorders | 1.97 | 0.00 | 0.44 |
| Respiratory, thoracic and mediastinal disorders | 1.48 | 0.00 | 0.00 |
| Metabolism and nutrition disorders | 1.43 | 0.00 | 0.27 |
| Gastrointestinal disorders | 1.39 | 3.69 | 2.93 |
| Hepatobiliary disorders | 1.16 | 0.79 | 0.62 |
| Skin and subcutaneous tissue disorders | 0.86 | 4.48 | 1.78 |
| Congenital, familial and genetic disorders | 0.60 | 0.40 | 0.00 |
| Vascular disorders | 0.60 | 1.05 | 0.00 |
| Blood and lymphatic system disorders | 0.44 | 0.00 | 0.00 |
| Infections and infestations | 0.35 | 0.00 | 0.00 |
| Reproductive system and breast disorders | 0.35 | 4.08 | 0.27 |
| Immune system disorders | 0.23 | 0.00 | 0.62 |
| Endocrine disorders | 0.19 | 0.40 | 0.53 |
| Pregnancy, puerperium and perinatal conditions | 0.16 | 0.00 | 0.00 |
| Ear and labyrinth disorders | 0.09 | 0.00 | 0.80 |
