# Supplementary material for: Safety of antidepressants commonly used in 6–17-year-old children and adolescents: A disproportionality analysis from 2014–2023 on the basis of the FAERS database
Source: PLoS One. 2025 Aug 13;20(8):e0330025. doi: 10.1371/journal.pone.0330025 (PMC12349705; doi:10.1371/journal.pone.0330025)
Supplement: S12 Table — (DOCX) [file pone.0330025.s012.docx]

**S12 Table. HLGT distribution of psychiatric disorders event signals.**

| **HLGT(High-Level Group Term)** | **Fluoxetine**  **(%)** | **Escitalopram**  **(%)** | **Sertraline**  **(%)** |
| --- | --- | --- | --- |
| Suicidal and self-injurious behaviours NEC | 33.55 | 38.11 | 34.08 |
| Sleep disorders and disturbances | 4.99 | 4.91 | 3.65 |
| Sexual dysfunctions, disturbances and gender identity disorders | 1.18 | 3.02 | 0.00 |
| Schizophrenia and other psychotic disorders | 0.20 | 1.13 | 1.01 |
| Psychiatric disorders NEC | 6.89 | 1.13 | 4.87 |
| Personality disorders and disturbances in behaviour | 6.70 | 3.02 | 7.71 |
| Mood disorders and disturbances NEC | 4.14 | 3.02 | 8.32 |
| Manic and bipolar mood disorders and disturbances | 2.17 | 3.77 | 2.64 |
| Impulse control disorders NEC | 0.33 | 0.00 | 0.81 |
| Eating disorders and disturbances | 0.66 | 0.00 | 0.00 |
| Disturbances in thinking and perception | 5.98 | 7.55 | 6.90 |
| Dissociative disorders | 0.72 | 0.00 | 0.00 |
| Developmental disorders NEC | 0.39 | 0.00 | 0.00 |
| Depressed mood disorders and disturbances | 8.40 | 11.32 | 4.87 |
| Deliria (incl confusion) | 5.12 | 0.00 | 5.48 |
| Communication disorders and disturbances | 0.33 | 0.00 | 0.00 |
| Cognitive and attention disorders and disturbances | 0.53 | 1.51 | 0.00 |
| Changes in physical activity | 4.01 | 3.02 | 4.06 |
| Anxiety disorders and symptoms | 13.72 | 13.96 | 10.14 |
| Psychiatric and behavioural symptoms NEC | 0.00 | 4.53 | 5.48 |
