# Supplementary material for: Safety of antidepressants commonly used in 6–17-year-old children and adolescents: A disproportionality analysis from 2014–2023 on the basis of the FAERS database
Source: PLoS One. 2025 Aug 13;20(8):e0330025. doi: 10.1371/journal.pone.0330025 (PMC12349705; doi:10.1371/journal.pone.0330025)
Supplement: S13 Table — (DOCX) [file pone.0330025.s013.docx]

**S13 Table. HLGT distribution of nervous system disorders event signals.**

| **HLGT(High-Level Group Term)** | **Fluoxetine**  **(%)** | **Escitalopram**  **(%)** | **Sertraline**  **(%)** |
| --- | --- | --- | --- |
| Movement disorders (incl parkinsonism) | 21.79 | 17.65 | 19.92 |
| Sleep disturbances (incl subtypes) | 1.70 | 0.00 | 1.24 |
| Neurological disorders NEC | 48.03 | 51.76 | 38.17 |
| Neuromuscular disorders | 12.01 | 9.41 | 13.28 |
| Mental impairment disorders | 6.59 | 3.53 | 2.07 |
| Spinal cord and nerve root disorders | 0.43 | 0.00 | 0.00 |
| Headaches | 2.23 | 0.00 | 17.01 |
| Seizures (incl subtypes) | 7.23 | 17.65 | 7.05 |
| Neurological disorders of the eye | 0.00 | 0.00 | 1.24 |
